# Fetus in Fetu: Report of Two Cases

**Published:** 2014-09-01

**Authors:** Ram Babu Goyal, Rahul Gupta, Girish Prabhakar, Rajan Dagla

**Affiliations:** 1 Professor and Head, Department of Pediatric Surgery, SMS Medical College, Jaipur; 2 Assistant Professor, Department of Pediatric Surgery, NIMS Medical College and University, Jaipur; 3 Professor, Department of Pediatric Surgery, SP Medical College, Bikaner; 4 Assistant Professor, Department of Pediatric Surgery, Adesh Medical College, Bhatinda, Punjab.

**Keywords:** Fetus-in-fetu, Monozygotic diamnionic twinning, Teratoma

## Abstract

Fetus-in-fetu (FIF) is a rare and interesting entity characterized byincorporation of a malformed, monozygotic, diamnionic parasitic twin into the body of other normal twin partner. FIF is differentiated from teratoma by its embryological origin, its unusual location in the retroperitoneal space and the presence of vertebral column (axis) often with appropriate arrangement of other organs or limbs around this axis. We report two cases of FIF. Our first case presented at 18 months, while second at 9 year of age. FIF derived their blood supply directly from aorta in both the cases.Our FIF had distinct fetoid features, well developed axial skeleton with a complete spinal column, trunk, intestinal loops, four limbs, well developed fingers and toes, male external genitalia and abundant scalp hairs. Their weightwas 600 grams and 800 grams, respectively. Postoperative period was smooth and on long-term follow up no evidence of recurrence was seen in both the patients.

## INTRODUCTION

Fetus-in-fetu(FIF) is an extremely rare congenital anomaly in which malformed, monozygotic diamnionic parasitic twin(one vertebrate fetus) is incorporated into the body of its twin partner and grows inside it, usually in the abdominal cavity, retroperitoneally (80%).[1]The term Fetus-in-fetu, first quoted by Lewis, but it was Johann Friedrich Meckel who first described it in early 19th century.[2] It was finally defined by Willis in 1953 as a mass containing a vertebral axis often with appropriate arrangement of other organs or limbs around this axis by which it is differentiated from the highly differentiated teratoma.[1] We present two cases of FIF and discuss their clinical profile, specimen findings, and long-term outcome.

## CASE REPORT

**Case 1**

An 18-month-old male child presented with a gradually increasing abdominal swelling. The child was anemic and malnourished. There was a large abdominal, rounded, non tender lump of variable consistency occupying the whole of the left half of the abdomen. Plain x-ray showed a mass effect on the left side of the abdomen with bones and calcification giving a suspicion ofteratoma.Barium enema (to rule out bowel involvement) revealed displacement of the colon with bones and calcification in the soft tissue mass (Fig.1). Threedimensional ultrasonography showed a well formed spine and arranged skeletal system.Computerised tomography (CT) scan of the patient revealed a large mass with variable solid and cystic consistency extending from left hypochondrium to pelvis and displacing the left kidney superolaterally and posteriorly along with spleen laterally. The internal structures of the mass showed fluid, fat, soft tissues and bony elements. Our preoperative suspicion was of FIF.

On exploration, mass enclosed in a complete sac was found in retroperitoneum, attached to surrounding structures and supplied by major vessels originating from aorta, renal artery and surrounding retroperitoneum. It was separated from surrounding structures and excised in toto. Postoperative recovery was normal.Excised specimen measured 10x8x7 cm and weighted 800 grams. Plain x-ray of the specimen showed well formed bony structures. On opening the sac, well formed FIF was seen. It had rudimentary head, and well developed thorax, abdomen, upper limbs with fingers, lower limbs with feet and toes and a well differentiated external genitalia (penis and scrotum). On further dissection of the fetus from dorsal side, a well developed vertebral column was found along with the rib cage (Fig.1). On dissecting the abdomen, it was found to be divided in two compartments, although with no definite recognizable intra-abdominal structures, but the embryological structures could be appreciated. Skull bone was seen, burr hole made and biopsy taken. Microscopic examination of the biopsy proved to be neurological tissue.

**Figure:**
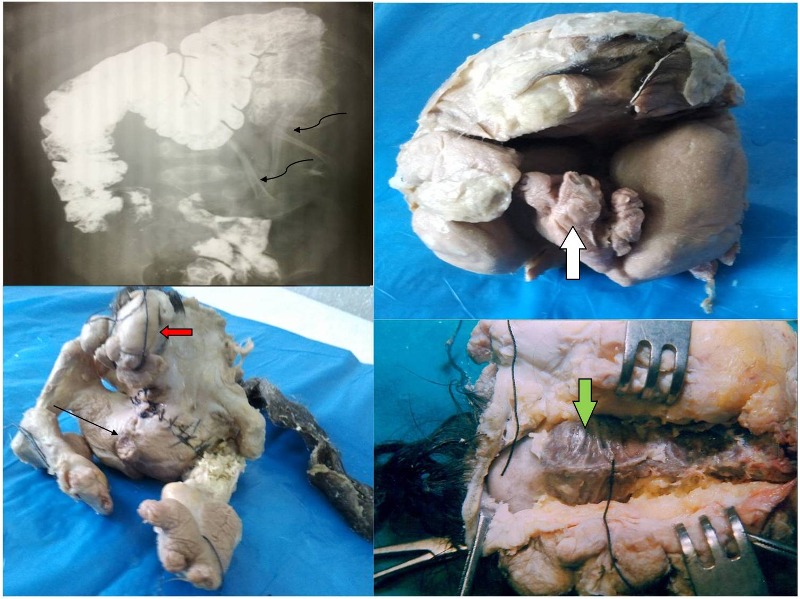
Figure 1:Top left-Barium enema showing displacement of colon due to soft-tissue mass. Bones and calcification in the mass is seen(curved black arrows). Top right- Well formed FIF seen after opening the sac(white arrow). Bottom left-FIF with rudimentary head(red arrow),well developed thorax, abdomen, upper and lower limbs and well developed external genitalia(straight black arrow).Bottom right- Dissection of the FIF from dorsal side showing-well developed vertebral column along with rib cage(green arrow).

**Case 2**

A 9-year-old young boy presented with pain abdomen for the last 3 months and appreciation of swelling in right hypochondrium for 2 months. On examination he was pale and there was fullness in right hypochondrium. Palpation of abdomen revealed a non tender, firm, spherical lump in right hypochondrium, not moving with respiration, extending upto the right lumbar and epigastric regions and just crossing midline. Its upper margins could not be palpated, though finger insinuation was possible between lump and right costal margin. The lower margins extended 6cms below the costal margin.

Plain skiagram of abdomen revealed soft tissue mass in the right upper abdomen. Ultrasound showed solid-cystic complex mass of 10x9 cms with multiple linear and rounded echogenic foci with distal acoustic shadows suggestive of bony parts seen superior to right renal region with mild hepatosplenomegaly. Intravenous urography revealed bilateral normal functioning kidneys. It was followed by Contrast Enhanced Computerised Tomography (CECT). A well defined rounded heterogeneous retroperitoneal mass of size 10.2 x 9.7 x 10.5 cm in right para-renal space, displacing right kidney downwards and well maintained fat planes between the mass and the adjacent structures, was present. The mass consisted of areas of fluid and fat attenuation, calcific densities to the shape of long bones and vertebrae along with some soft tissue attenuating areas with mild to moderate contrast enhancement of walls of mass.

On exploration, there was a large retroperitoneal mass in the right hypochondrium, densely adherent to diaphragm superiorly, inferiorly to right kidney, and large feeding vessels were seen entering into the tumor directly from aorta (Fig.2).The liver, C-loop of duodenum and the pancreas were pushed medially. Hepatobiliary tree was not involved by the mass. Inferior vena cava (IVC) was stretched and densely adherent to the anterior surface of the mass.By meticulous dissection mass was separated from surrounding structures, especially IVC. Feeding vessels were ligated and divided. Although the mass was densely adhered to surrounding organs, there was no damage to these structures.Mass was excised in toto and abdominal closure was undertaken.Postoperative recovery was normal.Gross specimen of the mass measured 12 x 10 x 10 cm and weighted 600 grams. On further dissection the specimen, a well formed FIF was delineated, embedded in thick pultaceous material. The fetus was anencephalic; well developed upper and lower limbs with well formed male external genitalia (Fig.2). FIF had 30 cm long hairs.

**Figure:**
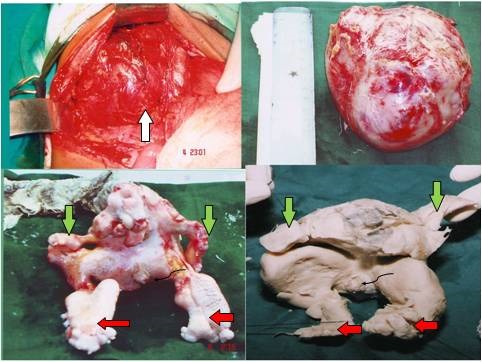
Figure 2:Top left- A large retroperitoneal mass(white arrow) in the right hypochondrium, densely adherent to diaphragm superiorly, inferiorly to right kidney. Inferior vena cava is stretched and densely adherent to the anterior surface of the mass. Top right- Specimen excised in toto. Bottom left and Bottom right- A well formed FIF. The fetus is anencephalic; well developed upper(green arrows) and lower limbs(red arrows) with well formed male external genitalia, long hair also seen.

Both patients were followed postoperatively and on long-term follow up there was no evidence of recurrence. They are doing well.

## DISCUSSION

The incidence of fetus-in-fetu is 1 per 5,00,000 births and less than 200 cases have been reported in the literature by year 2000.[3] It is usually evident in first year of life.[3] There’ve been few reported cases of FIF occurring later in life, as noted in our cases. Oldest documented patient with FIF was 47 year old man.[4]Common presentation (70%) is an asymptomatic abdominal mass, that is located in the upper retroperitoneum in 80% cases.[1,5,6] The other reported uncommon sites are urinary bladder, lesser sac, lower pole of kidney etc.[4-10]

FIF is mostly anencephalic, but in almost all cases vertebral column (91%) and limbs (82.5%) are present.[3] In presence of structures with an advanced grade of fetal development such as eyes, limb like processes, skin and colon, parts of central nervous system, genitalia, diagnosis of FIF is made even without the presence of spine.[9] The present case meets all the accepted criteria for a FIF.

FIF may be asymptomatic. Symptoms occur mainly due to its mass effect. [2-4] The preoperative diagnosis of FIF depends upon the radiological armamentarium.[3,5] The spinal column may be radiolucent and may be missed on radiological investigations.In recent years, magnetic resonance imaging has also been used to diagnose FIF.[8]

The fetus is typically lying in a complete sac composed of fibrous tissue (equivalent to the chorioamniotic complex) that contains some fluids (equivalent to the amniotic fluid) and is suspended by a pedicle. Two vessels are usually seen in this umbilical cord like pedicle.[2,3] In our case the sac was attached to the retroperitoneum similar to the placenta attached to the retroperitoneum. In our case and in most of the cases a single parasitic fetus was observed, although, multiple masses/ FIF, ranging from two to five have been found.[3]

Cases of reported FIF weighed between 1.2grams to 2000 grams.[1-10] The size of the fetus is related most likely to its blood supply. Fetuses with distinct vascular connections to the host are larger with better developed features. The blood supply of a FIF commonly comes mostly from the abdominal wall plexus in abdominal FIF, or any plexus where its sac is attached to the host’s wall.[6] 

FIF is a benign condition and complete surgical excision is curative. In addition, we consider a regular follow up, with the evaluation of the tumor markers and ultrasound examination, as one case of malignant recurrence after resection of FIF has been reported [7].

## Footnotes

**Source of Support:** Nil

**Conflict of Interest:** None declared

